# Signature of gene aberrant alternative splicing events in pancreatic adenocarcinoma prognosis

**DOI:** 10.7150/jca.48661

**Published:** 2021-03-31

**Authors:** Jun Yao, Yu-Chen Tang, Bin Yi, Jian Yang, Yun Chai, Ni Yin, Zi-Xiang Zhang, Yi-Jun Wei, De-Chun Li, Jian Zhou

**Affiliations:** 1 Department of General Surgery, the First Affiliated Hospital of Soochow University, Suzhou, Jiangsu, 215006, China.; 2 Pancreatic Disease Research Centre, The First Affiliated Hospital of Soochow University, Suzhou, Jiangsu, 215006, China.; 3 Department of Plastic Surgery, Suzhou Municipal Hospital, Suzhou, Jiangsu, 215006, China.; 4 Department of Oncology, the First Affiliated Hospital of Soochow University, Suzhou, Jiangsu, 215006, China.

**Keywords:** genomic analysis, alternative splicing, pancreatic adenocarcinoma, risk model, alternative splicing factors.

## Abstract

Alternative splicing (AS), as an effective and universal mechanism of transcriptional regulation, is involved in the development and progression of cancer. Therefore, systematic analysis of alternative splicing in pancreatic adenocarcinoma (PAAD) is warranted. The corresponding clinical information of the RNA-Seq data and PAAD cohort was downloaded from the TCGA data portal. Then, a java application, SpliceSeq, was used to evaluate the RNA splicing pattern and calculate the splicing percentage index (PSI). Differentially expressed AS events (DEAS) were identified based on PSI values between PAAD cancer samples and normal samples of adjacent tissues. Kaplan-Meier and Cox regression analyses were used to assess the association between DEAS and patient clinical characteristics. Unsupervised cluster analysis used to reveal four clusters with different survival patterns. At the same time, GEO and TCGA combined with GTEx to verify the differential expression of AS gene and splicing factor. After rigorous filtering, a total of 45,313 AS events were identified, 1,546 of which were differentially expressed AS events. Nineteen DEAS were found to be associated with OS with a five-year overall survival rate of 0.946. And the subtype clusters results indicate that there are differences in the nature of individual AS that affect clinical outcomes. Results also identified 15 splicing factors associated with the prognosis of PAAD. And the splicing factors ESRP1 and RBM5 played an important role in the PAAD-associated AS events. The PAAD-associated AS events, splicing networks, and clusters identified in this study are valuable for deciphering the underlying mechanisms of AS in PAAD and may facilitate the establishment of therapeutic goals for further validation.

## Introduction

Pancreatic adenocarcinoma is one of the most common malignant tumours, ranking fourth in cancer-related deaths, and the incidence of this cancer has increased gradually in recent years 1, 2. It is estimated that by 2030, pancreatic adenocarcinoma may exceed colorectal cancer and become the second most common cancer 3. Given the high mortality rate of pancreatic adenocarcinoma 4, achieving a better understanding of its risk factors is necessary to prevent pancreatic adenocarcinoma.

Currently, alternative splicing (AS) has been the proposed mechanism for tumourigenesis. AS is a key post-transcriptional regulatory mechanism and a major reason for enhancing transcriptome and proteome diversity 5. Emerging evidence suggests that aberrant alternative splicing is a common event in cancer development and progression and is closely related to such processes as proliferation, metastasis, and therapeutic resistance 6-8. Changes in the expression of key splicing factors can also result in changes in the AS of the target gene. For example, SRSF1 can direct the MYO1B gene to produce AS, which increases the tumourigenic potential of glioma cells via the PDK1 / AKT and PAK / LIMK pathways 9. CHERP and SR140 regulate the expression of UPF3A splices in CRC by forming protein complexes 10. In addition, the relevant alternative splicing event can even occur at the lncRNA level, and the lncRNA-PXN-AS1 splicing factor MBNL3 can produce different alternative splicing variants lncRNA-PXN-AS1-L and lncRNA-PXN- AS1-S, which bind to different parts of PXN mRNA, regulate PXN from two different directions and exert different biological functions 11.

Due to the high complexity and heterogeneity of pancreatic adenocarcinoma, the molecular mechanisms of its progression and early metastasis remain unclear 12, 13. Alternative splicing is one of the mechanisms leading to the complexity and effectiveness of disease progression. Therefore, it is particularly necessary to identify the link between aberrant alternative splicing and pancreatic adenocarcinoma. Currently, cancer-specific AS events are identified by comparing cancerous tissue with normal control tissue. Systematic profiling of prognostic AS features has been reported in lung 14, breast 15, rectal 16, and thyroid cancers 17. Currently, there is a lack of comprehensive research examining survival-related AS events in pancreatic adenocarcinoma.

In this study, we compared differentially spliced AS transcripts of pancreatic adenocarcinoma tissues and non-tumour tissues and genome-wide survival-related AS events in 171 patients with pancreatic adenocarcinoma. The RNA-Seq data and the corresponding clinical information of the PAAD cohort were analysed by DEAS and its splicing network by bioinformatics methods. A total of 45,313 AS events were identified, 1546 of which were differentially expressed AS events (DEAS). Nineteen DEAS events were found to be associated with OS with a five-year overall survival rate of 0.946. The regulatory relationship can be further predicted in the network constructed for the identified prognostic-related splicing factors and alternative splicing events. And the splicing factors ESRP1 and RBM5 played an important role in the PAAD-associated AS events. The PAAD-associated AS events and splicing networks we identified are of great value in deciphering the underlying mechanisms of AS in PAAD and may facilitate the establishment of therapeutic goals for further validation.

## Methods

### Alternative Splicing Event Curation from TCGA RNA-seq Data

**Figure 1:**
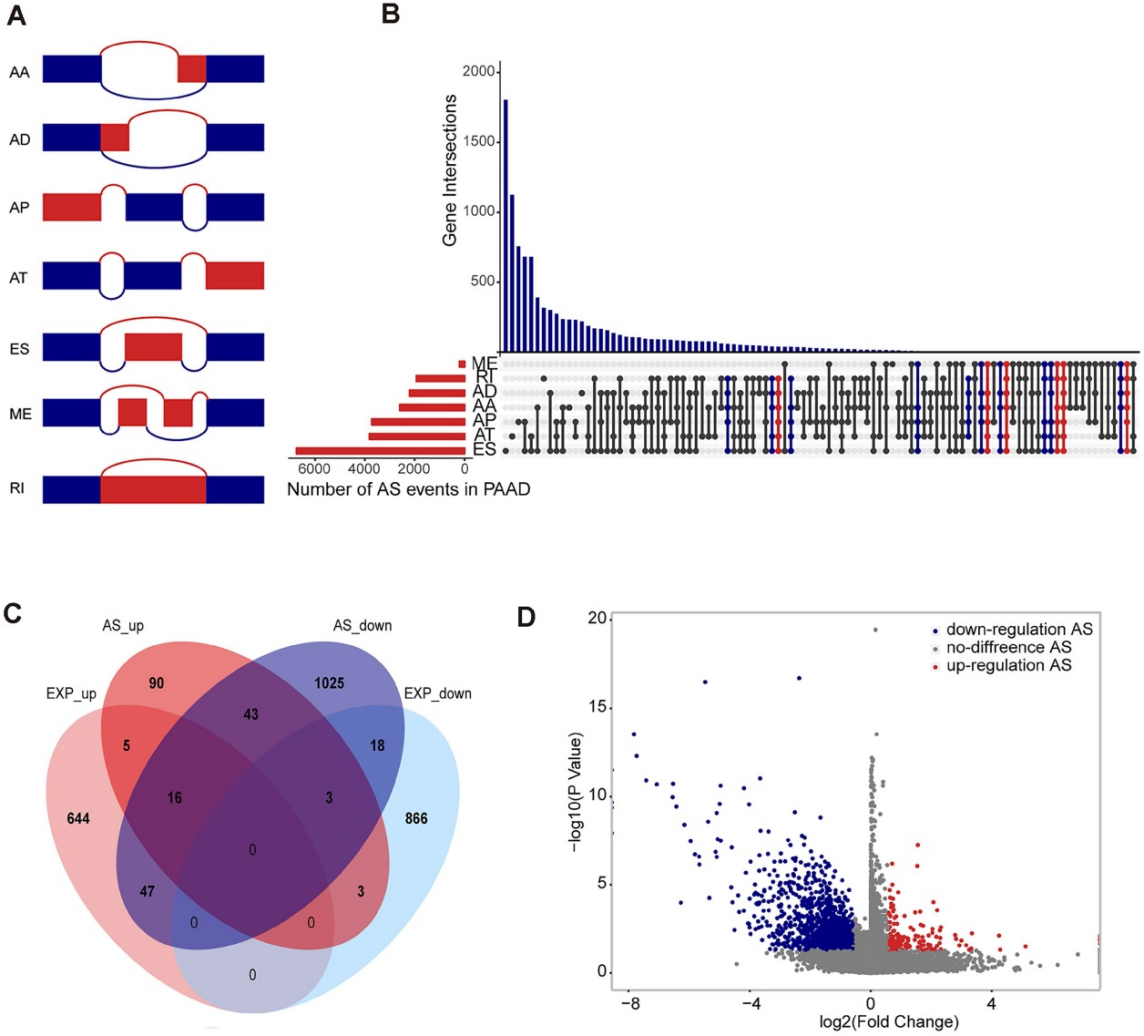
**Overview of seven types of alternative splicing and differentially spliced AS events analysis in pancreatic adenocarcinoma. (A)** Schematic diagram of the alternative splicing type. **(B)** Number of alternative splicing events of each type and distribution of genes involved in alternative splicing events. Five alternative splicing types are marked in blue, reaching 6-7 alternative splicing types marked in red. **(C)** Differentially alternative splicing events involve Venn plots of genes and differentially expressed genes, showing the number of their isomorphisms. **(D)** Volcanic maps of differentially alternative splicing events, where blue represents a downregulated differential alternative splicing event, red represents an upregulated alternative splicing event, and grey represents a non-differential alternative splicing event.

The TCGA Data Portal (https://portal.gdc.cancer.gov/projects) provides RNA-seq data for TCGA pancreatic adenocarcinoma. This study used the SpliceSeq tool to analyse AS profiles and assess the splicing patterns of mRNA in pancreatic adenocarcinoma patients. Percent splicing index (PSI), ranging from 0 to 1, are used to quantify AS events and calculate seven types of alternative splicing events: Exon Skip (ES), Mutually Exclusive Exons (ME), Retained Intron (RI), Alternate Promoter (AP), Alternate Terminator (AT), Alternate Donor site (AD), and Alternate Acceptor site (AA) (Figure 1A).

### Differentially Spliced AS Events Analysis

This study compared the analysed AS spectrum of pancreatic adenocarcinoma samples with normal adjacent cancer samples, identified differential alternative splicing events, and defined p-values less than 0.05, FC > 1.5 or FC < 2/3 as differential alternative splicing events. At the same time, the gene expression profiles of pancreatic adenocarcinoma samples and normal adjacent cancer samples were also analysed for differential expression, and differentially expressed genes were identified for further comparison.

### Alternative Splicing Events Statistics and GO and KEGG Enrichment Analysis

This study conducted a comprehensive statistical analysis of alternative splicing events. Traditionally, most studies use Venn diagrams to represent relationships between interactive sets, but alternative splicing events are divided into seven types. We use a more intuitive UpSet diagram for the overall display of alternative splicing events. At the same time, we use clusterProfiler to perform GO function enrichment analysis on survival-related alternative splicing events, to determine important related biological processes, cellular components and molecular functions, and use kobas database for KEGG analysis.

### Survival Analysis

This study included patients with pancreatic adenocarcinoma with complete clinical parameters and at least 90 days of overall survival (OS). For the PSI value of the AS event for each parameter, the patients were divided into two groups by median. Univariate Cox regression was used to identify associations between alternative splicing events and OS in each type, with p-values less than 0.05 being defined as survival-related alternative splicing events.

To remove any genes that might not be independent factors in prognostic predictors, multivariate Cox regression was applied to facilitate analysis of survival-related alternative splicing events in seven types. The prognostic risk score is determined by multiplying the linear combination of AS PSI by the corresponding regression coefficient (b) representing the associated weight. The regression coefficients were calculated from a multivariate Cox proportional hazards regression model. The risk score formula is as follows:


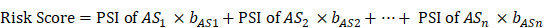


Finally, seven different types of candidate independent prognostic AS events were combined to construct a final prognostic predictor. In addition, the Kaplan-Meier curve of prognostic factors for pancreatic adenocarcinoma patients was compared within 5 years of OS. The chi-square test was used to compare the difference in survival status between the high-risk group and the low-risk group and to plot the ROC curve to predict the efficiency of each predictive model.

### Identify Clusters Associated with Prognosis and Molecular Subtypes

Alternative splicing events occur very differently at the individual level. To obtain robust classification, we used the unsupervised consensus approach implemented by Consussus Cluster Plus (R package) to identify molecular subtypes of pancreatic adenocarcinoma. Principal component analysis (PCA) were used to assess the optimal K. For the identified molecular subtypes, we start from the survival time to analyse the survival, identify the relationship between subtypes and survival, and further explore the relevant clinical information, trying to find other relationships between clinical information and molecular subtypes.

### Combined analysis of differential expression of prognostic factors and splicing factors

The GEO database verified the differential expression of 18 genes and 15 splicing factors, and 5 datasets (GSE22780, GSE27890, GSE32676, GSE16515, GSE15471) were selected for further analysis. Considering the limited number of normal tissues in the TCGA-PAAD database, we used the Genotype Tissue Expression Database (GTEx) combined with the TCGA database to verify the differential expression of 18 prognostic genes and 15 splicing factors. The GTEx database contains normal tissue samples from 54 human body parts, provides a data resource analysing and visualizing genomics data, thereby enabling researchers to explore and compare genetic alterations across samples.

### Splicing Correlation Network Construction

A list of 67 human splicing factors was created through hand-planned literature and database filtering 18. Expression of the splicing factor gene in the mRNA splicing pathway was derived from grade 3 mRNA-seq data in TCGA. Survival-related splicing factors were identified by one-way Cox regression analysis (p<0.05), and correlations between survival-related splicing factor gene expression and survival-related alternative spliced PSI values were analysed by Spearman's test. A choice of p-value less than 0.05 is defined as a significant correlation. The final interaction network between alternative splicing events and splicing factors was constructed using Cytoscape (3.6.0). At the same time, ClueGO (Cytoscape plug-in) was used for GO functional enrichment analysis and KEGG and Reactome pathway enrichment analysis to find significantly related GO terms or pathways. Gene set enrichment analysis (GSEA) in TCGA-PAAD data was performed using GSEA v3 software to evaluate the function of splicing factors ESPR1 and RBM5.

### IHC of ESRP1 and RBM5 thirty pairs of PC and adjacent normal tissues

PC and adjacent normal tissues were collected from the First Affiliated Hospital of Soochow University during the period of 2016 to 2019. The experiments were approved by the Ethical Committee of the First Affiliated Hospital of Soochow University and written informed consent was signed by each participant. All samples were incubated using rabbit polyclonal anti-ESRP1 antibody (catalog no. ab262886) and anti-RBM5 antibody (catalog no. AP70787) overnight at 4°C. The ESRP1 and RBM5 staining index were classified into four groups: score 0 (no staining), score 1 (0-20% of tumor cells stained), score 2 (20-50% of tumor cells stained) and score 3 (>50% of tumor cells stained). We defined that score > 1 indicated tumours with high ESRP1 (or RBM5) expression and score ≤ 1 indicated low/negative ESRP1 (or RBM5) expression 19.

### Statistical Analysis

All statistical analyses were performed using R/Bioconductor (version 3.5.1), reported p values < 0.05 were considered to be statistically significant, and p-values were bilateral.

## Results

### Integrated AS Events in Pancreatic Adenocarcinoma

In the 171 patients with pancreatic adenocarcinoma (Supplementary Table S1), 45,313 AS events associated with 10,622 genes were found. In detail, we detected 3,657 AAs out of 2,594 genes, 3,118 ADs in 2,210 genes, and 9,325 APs out of 3,724 genes, 8,733 ATs in 3,816 genes, 17,402 ESs in 6,749 genes, 205 MEs in 202 genes, and 2,872 RIs in 1,922 genes (Figure 1B). These results also indicate that a gene may have several types of mRNA splicing events, and one gene may exhibit up to five or even six to seven alternative splicing types, while ES is the dominant type because more than 1/3 of the AS type is an ES event.

### Differentially Spliced AS Events in Pancreatic Adenocarcinoma

Differential splicing analysis showed that 173 alternative splicing events and corresponding 160 genes were defined as up-regulated alternative splicing events in pancreatic adenocarcinoma, 1,373 alternative splicing events and corresponding 1,152 genes were downward adjustments (Figure 1C and D). In the differential expression analysis, 712 genes were defined as up-regulated differentially expressed genes in pancreatic adenocarcinoma, and 890 genes were defined as down-regulated differentially expressed genes. We compared the genes involved in the differentially alternative splicing event with the differentially expressed genes and found that the genes identified by the two groups have similarities but more obvious differences. Therefore, we believe that the analysis of the alternative splicing event can compensate for the deficiencies of differential expression analysis (Figure 1D).

### Survival Associated Alternative Splicing Events in Pancreatic Adenocarcinoma

**Figure 2:**
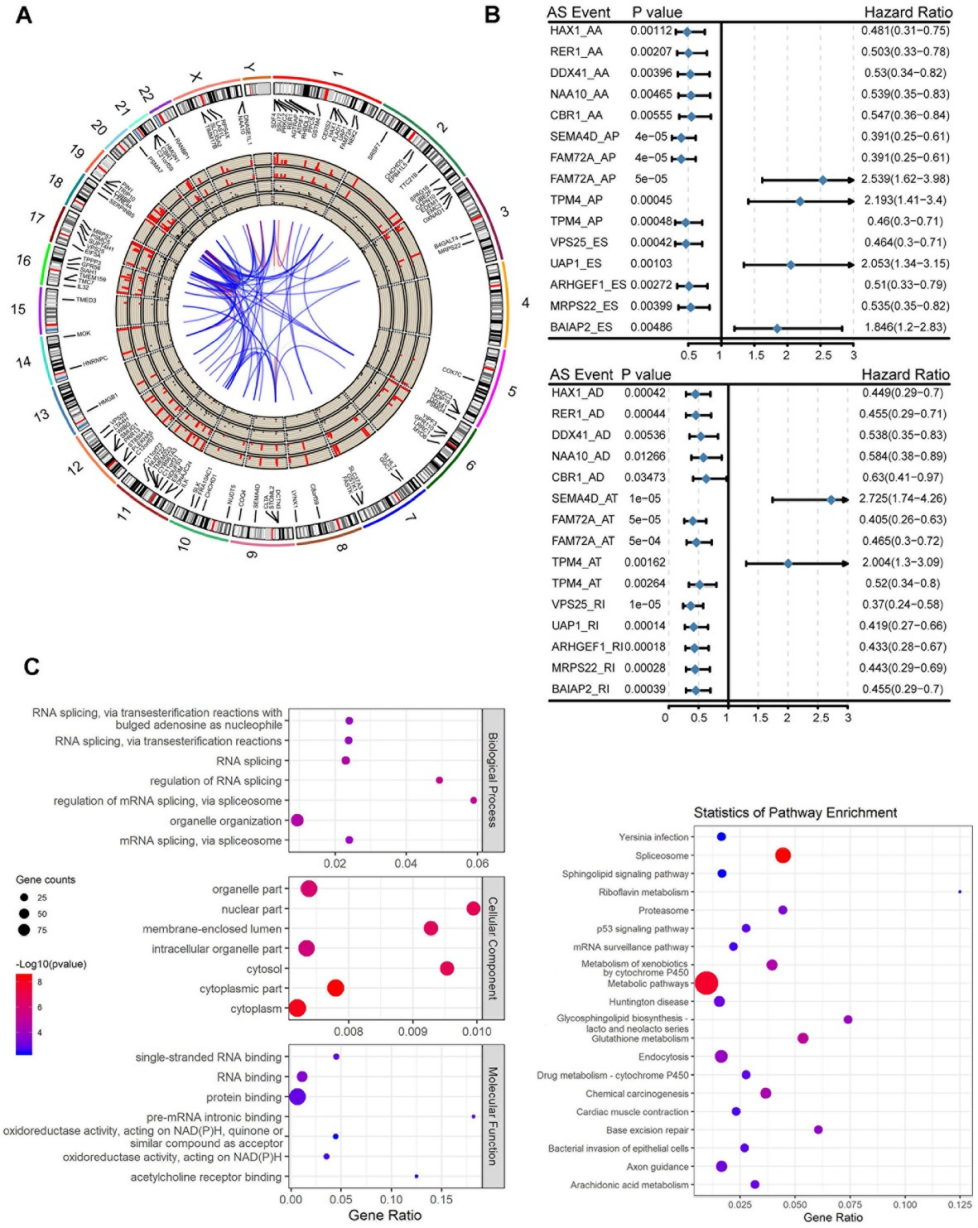
**Alternative splicing events statistics and GO functional enrichment analysis. (A)** The Circos diagram of the survival-related alternative splicing events and its related genes, the Circos panel from the outside to the inside, is expressed as follows: chromosome number, genomic axis, survival-related alternative splicing event-related genes, names of related genes, the number of related genes occurring in the overall alternative splicing events (1-10 (>10), and showing 1-10 different heights, over 10 calculations on time), the number of alternative splicing types of related genes in the overall events, the p value of the relevant gene in the difference analysis (expressed by the conversion value of -log10 (p-values), and the higher the height, the more significant the p value), the fold change value of the relevant gene in the difference analysis (where red represents upregulation and black represents downregulation), correlation between genes. **(B)** A forest map of the most important TOP5 survival-related alternative splicing events in each of the alternative splicing types after single factor Cox regression analysis. **(C)** GO and KEGG Enrichment analysis results of survival-related alternative splicing event-related genes after one-way Cox regression analysis.

Before investigating the prognostic value of the mRNA splicing event, a univariate survival test was performed to assess the relationship between clinical parameters and outcomes in TCGA pancreatic adenocarcinoma. In the pancreatic adenocarcinoma cohort, TNM staging (HR = 1.457, 95% CI: 1.064-1.994, P = 0.0189) and grade classification (HR = 1.421, 95% CI: 1.071-1.885, P = 0.0148) were significantly associated with OS. The results of this preliminary assessment indicate that the survival data of pancreatic adenocarcinoma samples in TCGA, although containing censored data, is informative and suitable for further molecular studies. To investigate the prognostic value of AS events in patients with pancreatic adenocarcinoma, we used univariate Cox regression analysis to assess the prognostic impact of differential alternative splicing events on patients with pancreatic adenocarcinoma. We detected a total of 130 survival-related alternative splicing events (p < 0.05) in differentially alternative splicing events. The Circos plot shows the details of survival-related AS events and their associated genes (Figure 2A) and takes six alternative splicing types (alternative splicing events associated with survival are not identified in the ME type). The most important survival-related alternative splicing events of TOP5 are plotted in the forest map (Figure 2B). Subsequently, GO functional enrichment analysis and KEGG analysis were performed on 116 genes involved in survival-related alternative splicing events (p < 0.05). The prognostic factors were enriched in cellular functions, such as RNA splicing, RNA splicing regulation, mRNA processing regulation, and the mitochondrial inner membrane, and enriched in metabolic pathway and spliceosome in KEGG pathway (Figure 2C).

### Prognostic Predictors for Pancreatic Adenocarcinoma

**Figure 3:**
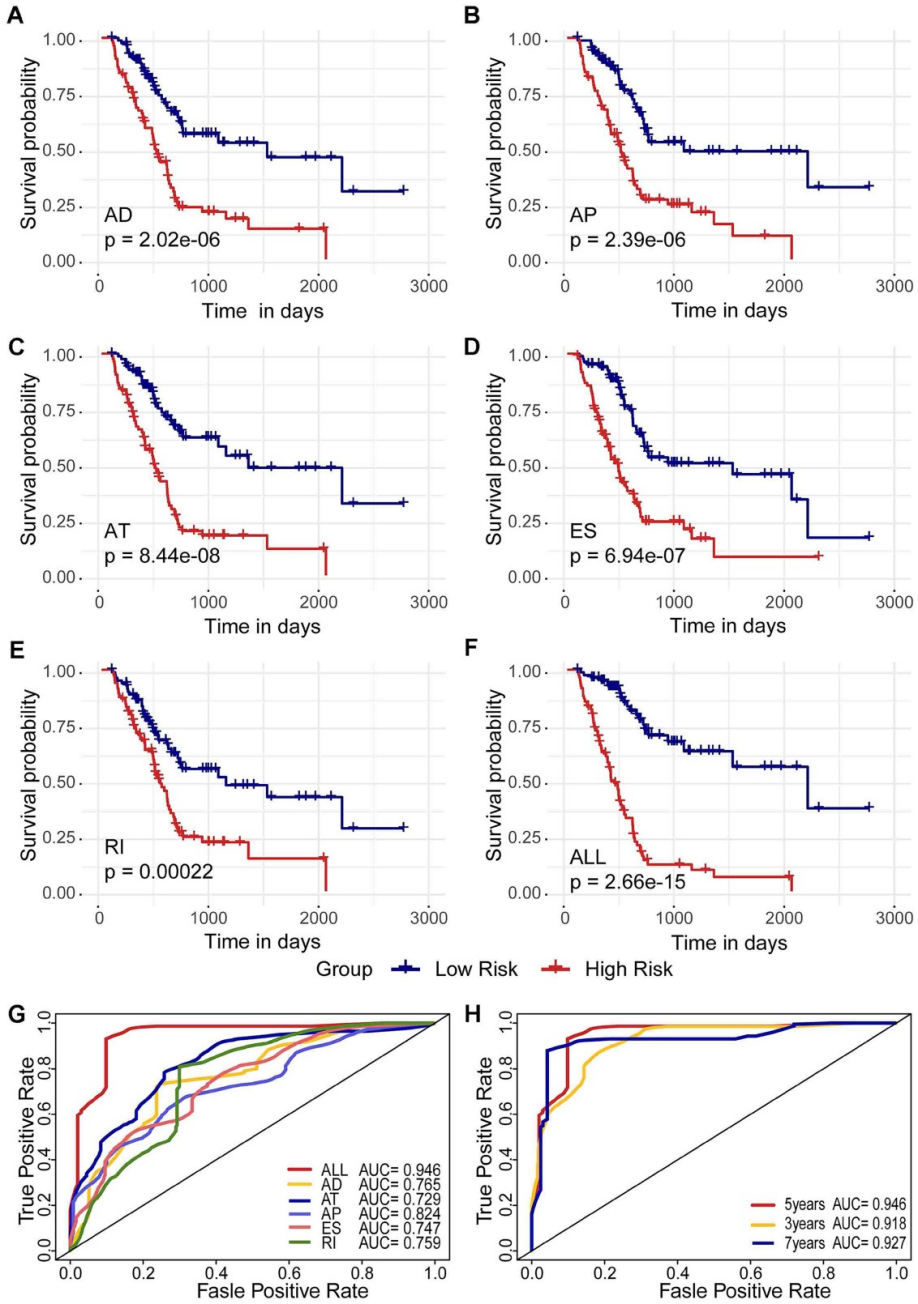
**Kaplan-Meier plots and ROC curves of predictive Kaplan-Meier plots and ROC curves of predictive factors in the TCGA pancreatic adenocarcinoma cohort. (A)-(E)** Kaplan-Meier curves plotted for prognostic models of each type of alternative splicing event. **(F)** Kaplan-Meier curves drawn from the prognostic model after integration of each type. The red line represents the high-risk group, and the blue line represents the low-risk group. **(G)** ROC curves for each type and post-integration alternative splicing event. **(H)** 3, 5, and 7 year ROC curves for alternative splicing events after integration.

To detect independent prognostic factors in patients with pancreatic adenocarcinoma, we selected survival-related alternative splicing events (p<0.01) as candidates and multivariate Cox regression analysis to identify independent prognostic factors in the six alternative splicing types retained (p < 0.05). Two independent prognostic factors associated with AD were obtained: three independent prognostic factors associated with AP, nine independent prognostic factors associated with AT, three independent prognostic factors associated with ES, and two independent prognostic factors associated with RI. Independent prognostic factors associated with AA were not identified (Table 1). Five different types of independent prognostic alternative splicing events were further combined to construct a final prognostic predictor. In our data analysis of each type of splicing pattern, the use of different types of alternative splicing events to construct a prognostic model demonstrated a significant ability to predict outcomes in patients with pancreatic adenocarcinoma, and these AS features may be a reliable predictor of prognosis in patients with pancreatic adenocarcinoma (Figure 3A-E, Supplementary Figure S1A-E). In particular, a prognostic model constructed from a single AP model showed better performance predicted by the five prognostic models (ROC = 0.824) (Figure 3G).

In addition, five different types of candidate independent prognostic AS events (19 total splicing events) were combined to construct a final prognostic predictor. It is worth noting that the final prognostic model showed better performance than each single type of splicing pattern in predicting (ROC = 0.946), as shown in Figure 3F (Supplementary Figure S1F) and G. It is conceivable that the final combined prognostic model is more efficient than other prognostic models. At the same time, we compare the ROC curves of the 3, 5 and 7 years of the final prognostic model. The model exhibited good predictive power (Figure 3H).

### Prognosis-Associated Molecular Subtype Cluster

**Figure 4:**
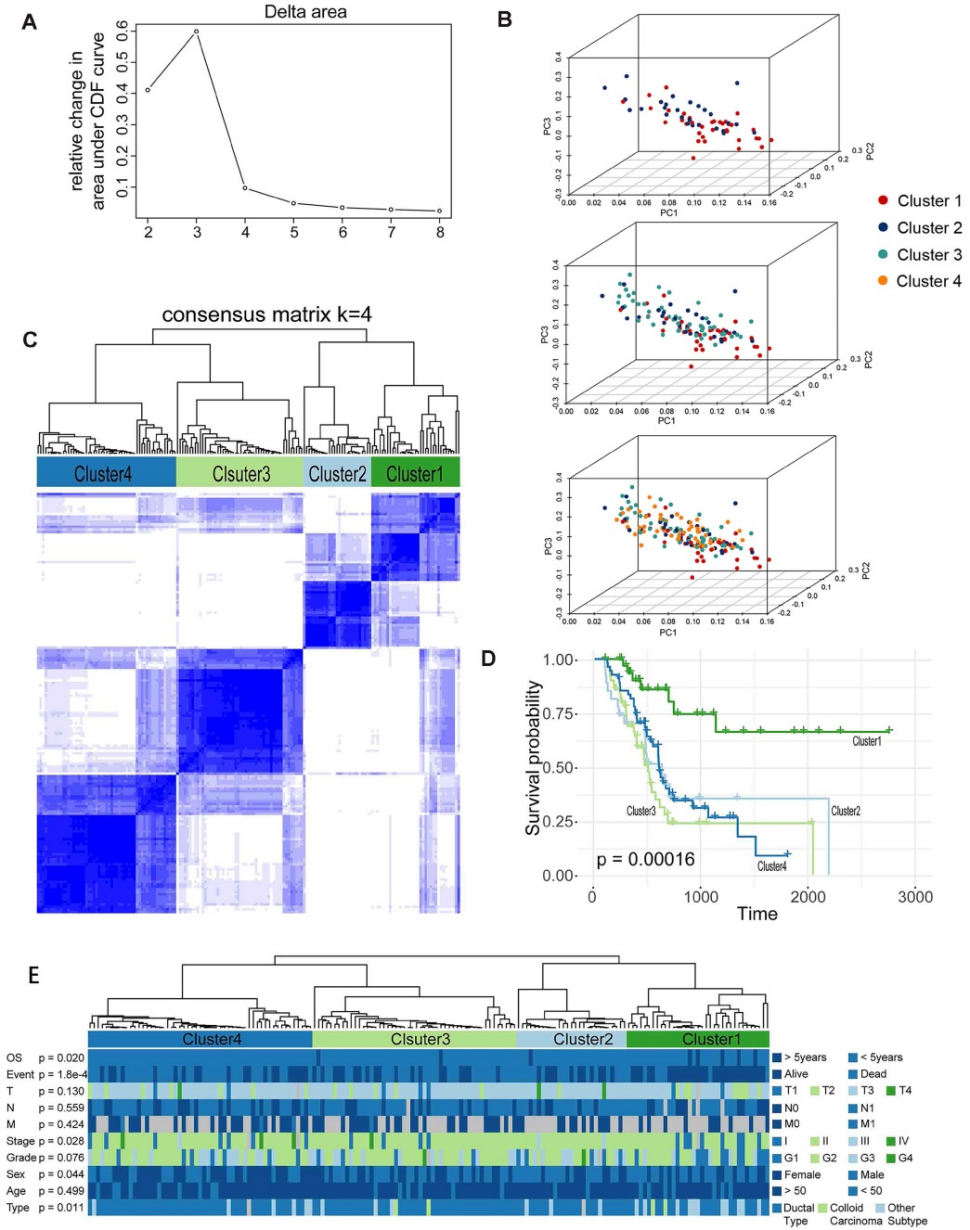
**Prognosis-associated molecular subtype cluster analysis. (A)-(B)** Statistical analysis of Elbow for different numbers of clusters (k = 2 to 8) and PCA analysis for K=4. **(C)** The consensus matrix heat map defines four sample clusters with consensus values ranging from 0 (white, samples never gathered together) to 1 (dark blue, samples are always clustered together). **(D)** Survival analysis in the identified four sample clusters. **(E)** The distribution of each clinical information in four sample clusters.

We further identified different AS patterns by unsupervised analysis of all samples based on 19 alternative splicing events associated with prognosis. By combining the Elbow method to determine the optimal number of clusters (Figure 4A), the PCA showed a relatively stable partitioning of the samples in the 4 clusters (Figure 4B), we finally determined four sets of samples as follows: C1 (n = 35, 21.0%), C2 (n = 27, 16.2%), C3 (n = 50, 29.9%), and C4 (n = 55, 32.9%) (Figure 4C).We then performed a Kaplan-Meier analysis to assess the relationship between clustering and prognosis. The results indicate that clusters are associated with different survival patterns, with Cluster3 correlating with poor outcomes in survival analysis (Figure 4D). At the same time, we further analysed the relevant clinical information and found that some related information was not randomly distributed, such as survival time (OS > 5 years or <5 years), survival status (Alive or Dead), and stage in four clusters. There was a significant difference in the histological type (chi-square test, p < 0.05) (Figure 4E). Therefore, we can also identify molecular subtype clusters associated with prognosis through alternative splicing events, and the results of the study indicate that there are differences in the nature of individual AS that affect clinical outcomes.

### Combined analysis of differential expression of AS-associated genes

In addition, in order to further identify the reliability of the prognostic factors, other databases besides TCGA were used to perform. Although the GEO and GTEx databases do not contain RNA-Seq information, they are of great significance for verifying the differential expression of prognostic factors. Five GEO datasets and TCGA combined GTEx datasets were used to verify the differential expression of prognostic factors. As shown in Table 2, most of the results show that these prognostic factors have expression differences in GEO and TCGA combined GTEx.

### Network of Survival-Associated AS Splicing Factors

**Figure 5:**
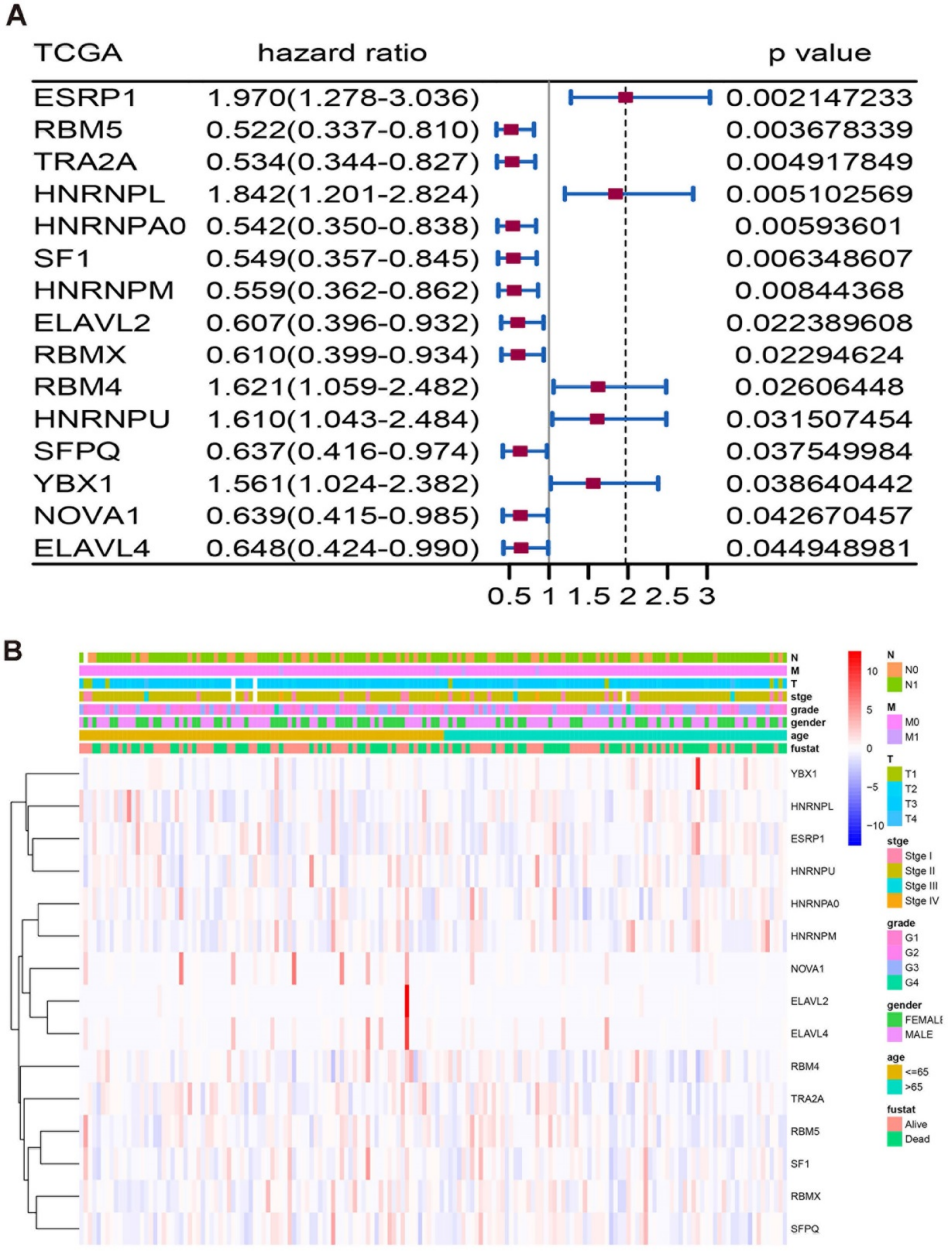
**Survival analysis and detail distribution of splicing factors. (A)** Forest plots visualizing the p-value of 15 splicing factors identified by survival analysis of TCGA. **(B)** The details of the 15 splicing factors in the TCGA cohort.

Through manual planning of literature and database screening, a list of 67 human splicing factors was created to further analyse the expression of splicing factor genes in the mRNA splicing pathway from TCGA. To determine which splicing factors are associated with AS events associated with survival in pancreatic adenocarcinoma, we performed a survival analysis of splicing factors, and the results showed that 15 splicing factors were significantly associated with overall survival (Figure 5A, Supplementary Table S2). The heat map shows the details of the 15 splicing factors in the TCGA cohort (Figure 5B).

**Figure 6:**
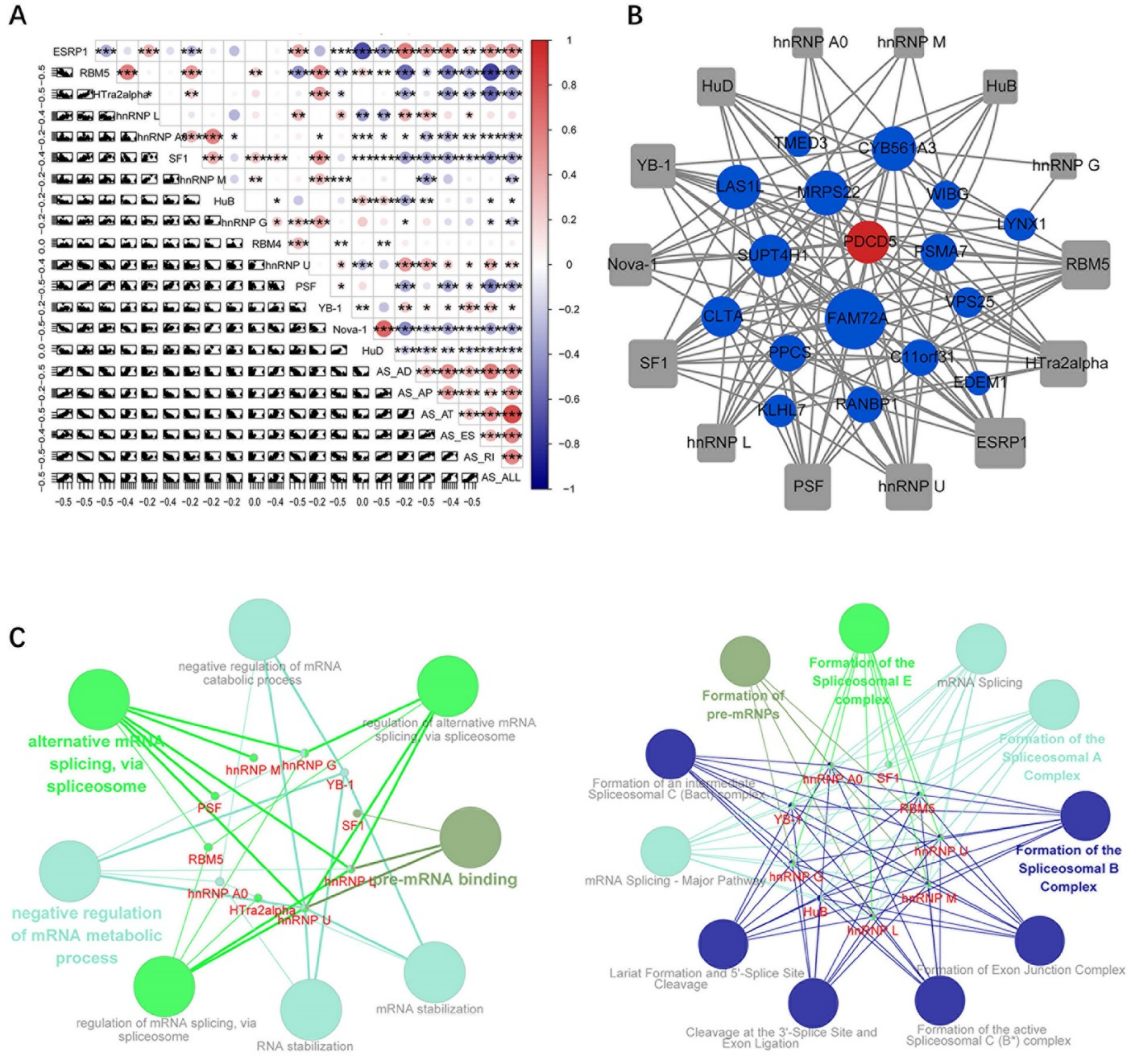
**Correlation analysis of splicing factors and prognosis-related AS predictors. (A)** Correlation analysis between splicing factors and AS prognostic predictors. The upper panel shows the correlation between correlation coefficient and splicing factor expression and PSI values of prognostic-related AS events. The size and colour of the circle represent the weight of the correlation coefficient, * p <0.05, ** p <0.01, *** p <0.001, and the scatter plot shows the correlation between the expression of the splicing factor and the PSI value of the survival-related AS event. **(B)** Alternative splicing network: the square node is the splicing factor, the circular node is the gene involved in the prognosis-related alternative splicing event, the blue node is the protective factor, and the red node is the risk factor. **(C)** Significantly enriched GO term, significantly enriched KEGG or Reactome pathway.

Through correlation analysis between the expression values of survival-related alternative splicing factors and the constructed prognostic models, we observed that the expression levels of most splicing factor genes were negatively correlated with the PSI of prognostic signals (Figure 6A). In addition, the Spearman test was used to investigate the correlation between the PSI values of the most important AS events and the expression of survival-related splicing factors. Among these factors, 14 survival-related splicing factors (grey points) were significantly associated with 18 survival-related AS events (corresponding to 17 genes, 16 protective factors (blue dots) and 1 risk factor (red dots)), which constituted 129 line alternative splicing networks with interactions (Figure 5B). Subsequently, ClueGO was used to perform enrichment analysis on the relevant genes in the network, and the significantly enriched GO term and related pathways were found. The ClueGO enrichment results are closely related to the pre-transcriptional regulation of mRNA, such as pre-mRNA binding, mRNA stabilization, alternative mRNA splicing (via spliceosome), mRNA splicing, and formation of the spliceosomal E complex (Figure 5C).

In addition, we also used five GEO datasets and the TCGA combined GTEx dataset to verify the differential expression of splicing factors, and almost all splicing factors have obvious expression differences in the GTEx combination of GEO and TCGA (Table 3).

### Specific analysis of splicing factors ESRP1and RBM5

**Figure 7:**
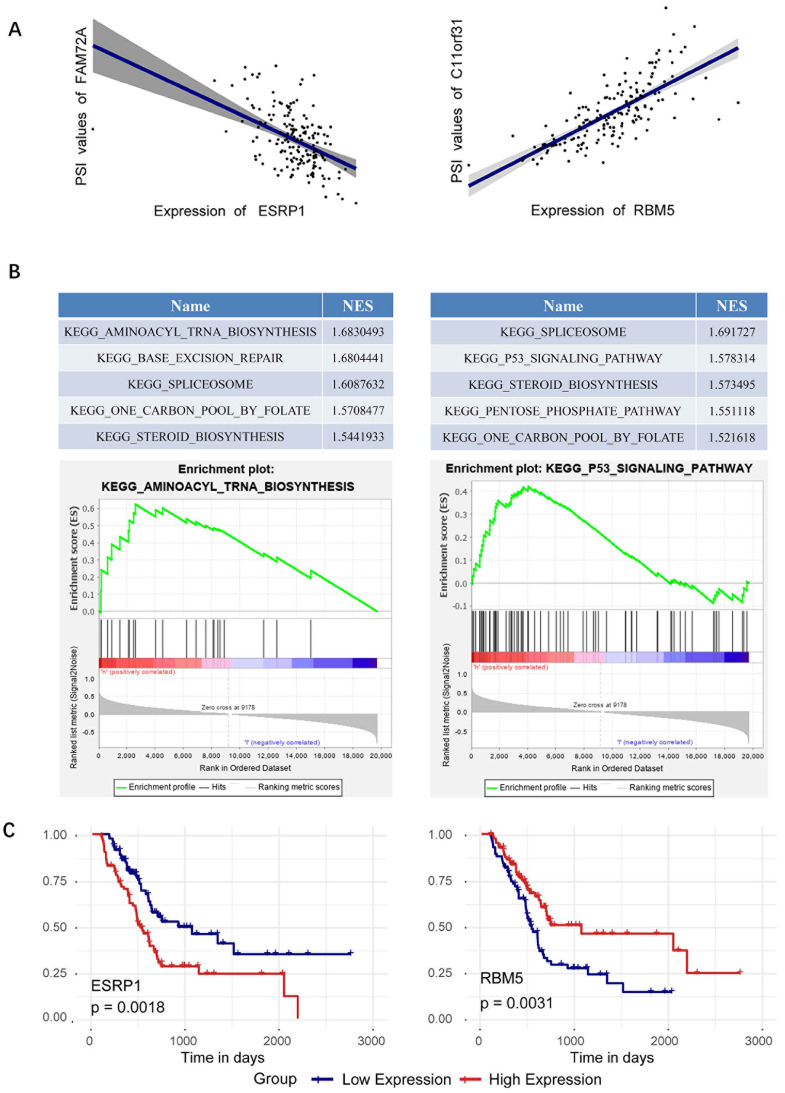
**Specific analysis of splicing factors ESRP1and RBM5. (A)** ESRP1 is negatively correlated with FAM72A, and RBM5 is positively correlated with C11orf31. **(B)** GESA analysis of splicing factors ESRP1and RBM5. **(C)** Survival curves of the identified survival-associated splicing factors ESRP1 and RBM5, with the red line representing the high expression group and the blue line representing the low expression group.

Since splicing factors ESRP1and RBM5 perform best in survival analysis, we focus on analyzing the respective regulatory relationships between ESRP1 and RBM5 in the SF-AS network. The network revealed that the correlation between the splicing factor ESRP1 and alternative splicing events was 14-fold, and the correlation between the splicing factor RBM5 and alternative splicing events was also 13-fold (Figure 6B). Specifically, in the AT splicing type, the splicing factor ESRP1 is negatively correlated with FAM72A, and the splicing factor RBM5 is positively correlated with C11orf31 (Figure 7A). GESA enrichment analysis results show that ESRP1 and RBM5 are significantly enriched in splice some pathway, while ESRP1 plays an important role in the aminoacyl tRNA biosynthesis pathway, and RBM5 participates in the P53 signaling pathway (Figure 7B). Finally, we plotted the prognostic curve of ESRP1 and RBM5 in TCGA (Figure 7C). And a survival analysis based on ESRP1 or RBM5 signature was further performed in the subgroup of patients with different clinical variables in the TCGA cohort. For the TCGA cohort, after stratifying the clinicopathological characteristics by gender, age, tumor size, pathological stage and AJCC stage, ESRP1 showed significance in age(<=60), male, and AJCC stage I+Ⅱ (Supplementary Figure S2). RBM5 showed significance in age (>60), male, pathologic stage I+Ⅱ, AJCC stage Ⅱ+Ⅲ+Ⅳ and Tumor size T1+T2 (Supplementary Figure S3).

### Immunohistochemistry (IHC) of splicing factors ESRP1 and RBM5

**Figure 8:**
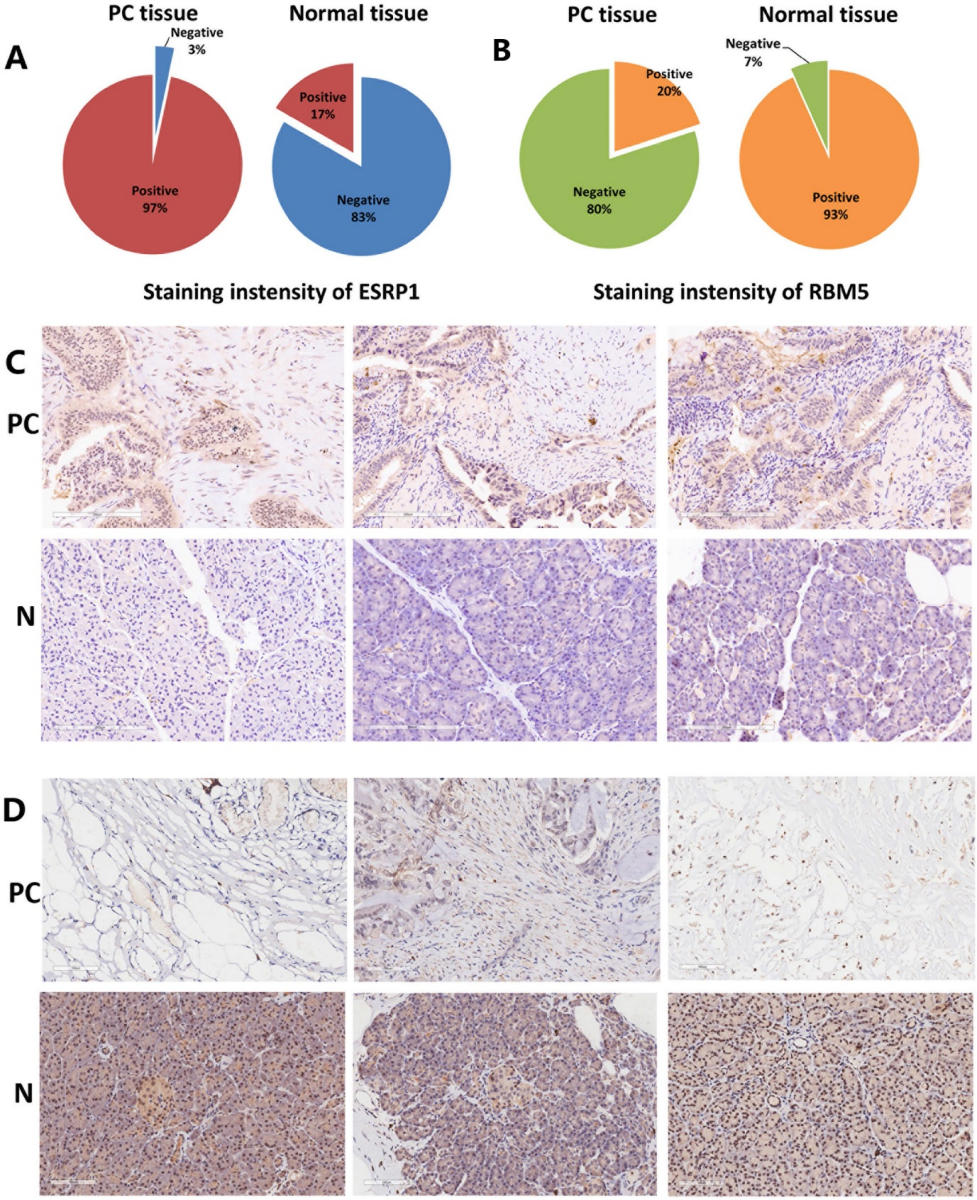
**Representative images for immunostaining of splicing factors ESRP1and RBM5. (A)** Pie chart reports the numbers of PC tissues versus normal tissues with 'positive' or 'negative' ESRP1 staining. **(B)** Pie chart reports the numbers of PC tissues versus normal tissues with 'positive' or 'negative' RBM5 staining. **(C)** Immunostaining for ESRP1 of PC tissues and normal tissues (x200). **(D)** Immunostaining for RBM5 of PC tissues and normal tissues (x200).

Immunostaining images of ESRP1 and RBM5 in thirty pairs of PC and adjacent normal tissues were displayed in Figure 8. ESRP1 and RBM5 were primarily expressed in cytoplasm. The positive staining of ESRP1 was detected in the majority of PC tissues (n=30, positive 97%) but less frequently in adjacent normal tissues (n=30, positive 17%). Contrarily, the positive staining of RBM5 was observed weaker in PC tissues (n=30, positive 20%) but stronger in adjacent normal tissues (n=30, positive 93%). IHC proved that ESRP1 was up-regulated in PC compared with adjacent normal tissues, and the RBM5 was down-regulated in PC tissues. The experimental results of immunohistochemistry support the analysis content of splicing factors ESRP1 and RBM5 in the bioinformatics database.

## Discussion

It has been previously shown that alternative splicing events contribute to cancer development and progression, and all processes of cancer (such as angiogenesis, cell proliferation, invasion, and immune response) are associated with alternative splicing of key genes 6, 7, 20. For example, the effect of CRKL on alternative splicing may be significantly associated with tumourigenesis in cervical cancer 21. SRSF3 promotes the expression of splice-like GRα which, in turn, regulates the migration and migration of breast cancer cells by RACK1 22. HNRNPLL regulates AS of CD44 to promote proliferation and metastasis of colorectal cancer 23. Changes in splicing factor expression that regulate alternative splicing events have also been identified in lung cancer, affecting such proteins as QKI, RBM4, RBM5, RBM6, RBM10 and SRSF1, which are important splicing factors in lung cancer alternative splicing events 24. Even more notable is that similar to the aforementioned lncRNA-PXN-AS1 11, the isoform due to alternative splicing can have the opposite effect on cancer. For example, in oral squamous cell carcinoma, depending on whether exon 23 is missing, STAT3 produces two variants by alternative splicing. STAT3α encodes a full-length oncogenic STAT3α protein, while STAT3β encodes a tumour-suppressed STAT3β protein. Specifically, PCBP1 is a key splicing factor that regulates alternative splicing of STAT3 exon 23 and promotes the transformation from oncogenic STAT3α to tumour suppressor STAT3β 25. Downregulation of ITSN1-L expression and upregulation of ITSN1-S expression may be one of the mechanisms of glioma proliferation and invasion, suggesting that regulation at the level of splicing may be an effective therapeutic strategy 26.

Alternative splicing events affecting pancreatic adenocarcinoma have also been found in many confirmatory studies. For example, two alternative splicing isoforms of RHAMM are RHAMMA and RHAMMB. RHAMMB is significantly upregulated in pancreatic adenocarcinoma liver metastasis, which promotes PNET metastasis via EGFR signalling, whereas RHAMMA does not 27. Compared to WT-MUC4, MUC4/X enhances panc-1 cell proliferation, invasion and adhesion and promotes pancreatic tumourigenesis via the integrin-β1/FAK/ERK signalling pathway 28. In the study of pancreatic adenocarcinoma treatment strategies, it was found that PKM2 splicing and expression are functionally related to the resistance of gemcitabine and cisplatin. Switching splicing of PKM1 by ASO transfection can enhance cell drug sensitivity 29. AS events of FGFR-2, SPAR, COL6A3 in pancreatic adenocarcinoma have also been previously reported 30-32, and an increase in AS of the KLF6 cancer suppressor gene is associated with pancreatic adenocarcinoma prognosis and tumour grade 33. Therefore, a comprehensive understanding of the AS model is critical to the future treatment strategy for cancer. The emergence of RNA-Seq technology and the cancer genome database has become a new tool for studying AS events in pancreatic adenocarcinoma, helping to identify new therapeutic and molecular targets for pancreatic adenocarcinoma 34, 35.

In this study, we compared differentially spliced AS transcripts of pancreatic adenocarcinoma tissues and non-tumour tissues and genome-wide survival-related AS events in 171 patients with pancreatic adenocarcinoma. A total of 45,313 AS events were identified, including 1546 differentially alternative splicing events, suggesting that alternative splicing is a common process in pancreatic adenocarcinoma. Specifically, we analysed seven types of splicing patterns of AA, AD, AP, AT, ES, RI, and ME and found that ES is the most important splicing method, which may also be responsible for the biological activity and protein complication of pancreatic adenocarcinoma 36. Furthermore, we found that genes for prognosis-related AS events are involved in multiple metabolic pathways involved in cancer cell biology and interact closely with each other. And by multivariate Cox regression analysis, 19 DEAS events were found to be associated with the prognosis of pancreatic adenocarcinoma. The prognostic predictors established in the study performed well in PAAD with a five-year overall survival rate of 0.946, representing a potentially reliable predictor of AS events in PAAD patients. Prognostic genes include LAS1L, SUPT4H1, FAM72A, C11orf31 and MRPS22, which play key roles in the biological mechanisms of cancer. At the same time, these prognostic genes may also participate in the process of cancer biology through means other than alternative splicing. For example, LAS1L and SENP3, as components of the MLL1-WDR5 super complex, regulate pancreatic adenocarcinoma gene transcription by affecting chromatin remodelling 37. MRPS22 is thought to be a potential driver involved in DNA replication, mismatch repair, p53 signalling pathway and cancer-associated signalling pathways 38. C11orf31 is involved in the regulation of drug resistance and cellular reactivity of ascorbic acid in a variety of cancer cells 39.

At a deeper level, we identified 15 splicing factors associated with the prognosis of PAAD, including the universal splicing factor SFs and the hnRNP family 40, 41. A combination of splicing factor expression and alternative splicing events and related networks are constructed to reveal the underlying mechanisms of the AS pathway. We found a close relationship between 15 splicing factors and 17 splicing genes, while the splicing factors ESRP1 and RBM5 performed well in the network. Studies have shown that ESPR1 and RBM5 play an important role in the event of alternative splicing in cancer 42, 43, and the results of GESA also show that ESRP1 and RBM5 are significantly enriched in the spliceosome pathway. In addition, evidence from experiments shows the interaction between ESRP1 and hnRNPM is related to EMT and breast cancer subtyping 44. ESRP1 can regulate the expression of FGFR-2 isoform FGFR-2IIIb, attenuates cell growth, migration, invasion and metastasis, and is a prognostic factor for pancreatic adenocarcinoma 45. Subsequent analysis of subgroups of patients with different clinical variables in the TCGA cohort showed that ESRP1 and RBM5 parts showed prognostic differences, but this does not mean that the results are biased, which may be due to data bias, population susceptibility and cancer heterogeneity. In short, we used databases such as TCGA, GEO, GTEx and immunohistochemical experiment to focus on analyzing the complications of alternative splicing on pancreatic cancer, and believe that we can have an in-depth understanding of the regulatory mechanisms of PAAD patients.

In summary, we found differential splicing of AS events between PAAD and normal tissues and established a model to demonstrate that survival-associated AS features can be used to predict prognosis in PAAD patients. The SF-AS network suggests a new potential mechanism in the carcinogenesis of PAAD, and the splicing factors ESRP1 and RBM5 played an important role in the PAAD-associated AS events. The systematic study of alternative splicing mechanisms provides a new direction for the treatment strategy of pancreatic adenocarcinoma.

## Supplementary Material

Supplementary figures and tables.

## Figures and Tables

**Table 1:** Independent prognostic factors in alternative splicing types with pancreatic cancer (p < 0.05).

AS_ID	Gene	Type	P-value	HR	Low 95%CI	High 95%CI
ID_89320	LAS1L	AD	0.0031	0.0001123	2.71E-07	0.0464984
ID_42666	SUPT4H1	AD	0.00821	1.23E-05	2.80E-09	0.0537494
ID_9575	FAM72A	AP	0.00014	0.0771458	0.0206222	0.2885959
ID_22286	WIBG	AP	0.0812	60.139324	0.6017212	6010.6543
ID_48878	PDCD5	AP	0.89371	0.8453955	0.0719646	9.9311824
ID_85365	LYNX1	AT	4.73E-05	0.2493761	0.1277371	0.4868471
ID_15925	C11orf31	AT	0.00039	3.80E-21	1.94E-32	7.45E-10
ID_32153	TMED3	AT	0.00165	1.28E+267	5.91E+100	Inf
ID_9577	FAM72A	AT	0.00549	0.0860268	0.015227	0.4860172
ID_63032	EDEM1	AT	0.00859	3.673E+09	270.12343	4.995E+16
ID_2074	PPCS	AT	0.02444	4.34E-12	5.50E-22	0.0342591
ID_78951	KLHL7	AT	0.03529	3.99E-13	1.14E-24	0.1398364
ID_86327	CLTA	AT	0.04937	5.365E+14	1.0987634	2.62E+29
ID_60044	PSMA7	AT	0.11692	213902157	0.0082526	5.544E+18
ID_67027	MRPS22	ES	0.00097	1.52E-09	8.76E-15	0.0002621
ID_680	AGTRAP	ES	0.00116	1.436E+11	26774.479	7.703E+17
ID_41127	VPS25	ES	0.0022	1.33E-35	6.37E-58	2.79E-13
ID_16163	CYB561A3	RI	0.00051	0.000185	1.45E-06	0.0235353
ID_61140	RANBP1	RI	0.2219	0.0348577	0.0001597	7.6079761

**Table 2:** The 18 genes in alternative splicing types differentially expressed with pancreatic cancer.

Gene	Five GEO datasets			TCGA combined GTEx datasets		
	log_2_^FC^	*P* value	Adjusted* p* value	log_2_^FC^	*P* value	Adjusted* p* value
*AGTRAP*	28.41	0.060065	0.120577	72921.16	6.87E-07	8.83E-07
*C11orf31*	382.54	0.052535	0.120577	41386.45	3.55E-13	6.39E-13
*CLTA*	248.16	0.023342	0.120577	156514.22	2.58E-20	5.16E-20
*CYB561A3*	203.78	0.042529	0.120577	-52884.05	3.10E-49	6.97E-49
*EDEM1*	103.37	0.025767	0.120577	-324291.22	7.27E-69	1.87E-68
*FAM72A*	23.31	0.026911	0.120577	-130.57	0.639447	0.677062
*KLHL7*	39.13	0.080385	0.120577	6569.78	7.97E-10	1.19E-09
*LAS1L*	17.24	0.263855	0.279376	-98400.06	2.42E-109	8.71E-109
*LYNX1*	23.06	0.125112	0.150135	366.73	0.9522	0.9522
*MRPS22*	129.97	0.12067	0.150135	-6471.30	0.000548	0.000617
*PDCD5*	6.84	0.656805	0.656805	24458.05	5.70E-08	7.90E-08
*PPCS*	240.21	0.054586	0.120577	26130.70	1.81E-06	2.17E-06
*PSMA7*	344.63	0.017642	0.120577	682454.68	6.52E-174	5.87E-173
*RANBP1*	26.76	0.071818	0.120577	225448.40	2.50E-142	1.50E-141
*SUPT4H1*	82.57	0.076974	0.120577	-912175.00	2.29E-200	4.13E-119
*TMED3*	46.29	0.122238	0.150135	-64975.98	2.15E-11	3.52E-11
*VPS25*	50.40	0.06168	0.120577	115857.38	4.38E-87	1.31E-86
*WIBG*	30.67	0.184132	0.207148	107219.64	1.35E-125	6.07E-125

**Table 3:** The 15 splicing factors differentially expressed in pancreatic cancer.

Gene	Five GEO datasets			TCGA combined GTEx datasets		
	log_2_^FC^	*P* value	Adjusted* p* value	log_2_^FC^	*P* value	Adjusted* p* value
*ELAVL2*	7.75	6.38E-12	7.98E-12	619.73	1.93E-05	2.41E-05
*ELAVL4*	-65.99	4.09E-35	3.07E-34	2675.26	0.003551	0.004098
*ESRP1*	407.94	1.29E-25	3.86E-25	73705.38	6.95E-17	1.16E-16
*HNRNPA0*	186.84	1.12E-14	1.53E-14	68273.36	4.83E-42	1.03E-41
*HNRNPL*	-59.93	9.84E-12	1.14E-11	43150.39	2.33E-17	4.36E-17
*HNRNPM*	12.71	0.099515	0.099515	111358.08	2.73E-61	6.83E-61
*HNRNPU*	-98.38	2.23E-07	2.39E-07	19590.14	0.353961	0.379244
*NOVA1*	28.82	8.53E-26	3.20E-25	388.42	0.654919	0.654919
*RBM4*	69.88	4.53E-26	2.26E-25	98766.58	1.70E-86	5.09E-86
*RBM5*	-414.03	5.09E-37	7.63E-36	-77494.35	1.25E-86	4.67E-86
*RBMX*	30.34	4.02E-23	1.01E-22	-171121.44	1.92E-100	1.44E-99
*SF1*	-89.17	7.94E-23	1.70E-22	-91532.75	5.38E-15	8.07E-15
*SFPQ*	330.84	2.40E-19	4.51E-19	-181385.34	1.94E-08	2.65E-08
*TRA2A*	113.27	1.92E-15	2.87E-15	-1377268.74	3.74E-88	1.87E-87
*YBX1*	-698.48	8.45E-18	1.41E-17	1480274.26	2.32E-118	3.49E-117

## Abbreviations

AS: Alternative splicing
PAAD: Pancreatic Adenocarcinoma
DEAS: Differentially Expressed Alternative Splicing Events
TCGA: The Cancer Genome Atlas
PSI: Splicing Percentage Index
ES: Exon Skip
ME: Mutually Exclusive Exons
RI: Retained Intron
AP: Alternate Promoter
AT: Alternate Terminator
AD: Alternate Donor
AA: Alternate Acceptor
OS: Overall Survival
ROC: Receiver Operating Characteristic
KEGG: Kyoto Encyclopedia of Genes and Genomes Pathway
GEO: Gene Expression Omnibus database
GTEx: Genotype-Tissue Expression
