# Supplementary material for: Signature of gene aberrant alternative splicing events in pancreatic adenocarcinoma prognosis
Source: J Cancer. 2021 Mar 31;12(11):3164–79. doi: 10.7150/jca.48661 (PMC8100795; doi:10.7150/jca.48661)
Supplement: Supplementary file 1 — Supplementary figures and tables. [file jcav12p3164s1.pdf]

Supplementary Figure S1. The prognostic models with single prognosis-relevant splicing events

(A) Alternate Donor site; (B)Alternate Promoter, (C) Alternate Terminator,(D) Exon Skip,(E) Retained Intron, (F) Kaplan-Meier curves drawn from the prognostic model after integration of each type.

Supplementary Figure S2. Survival analysis based on ESRP1 signature was further performed in the subgroup of patients with different clinical variables in the TCGA cohort. (A) Age, (B) Gender, (C) Pathologic stage, (D) AJCC stage, and (E) Tumor size.

Supplementary Figure S3. Survival analysis based on RBM5 signature was further performed in the subgroup of patients with different clinical variables in the TCGA cohort. (A) Age, (B) Gender, (C) Pathologic stage, (D) AJCC stage, and (E) Tumor size.

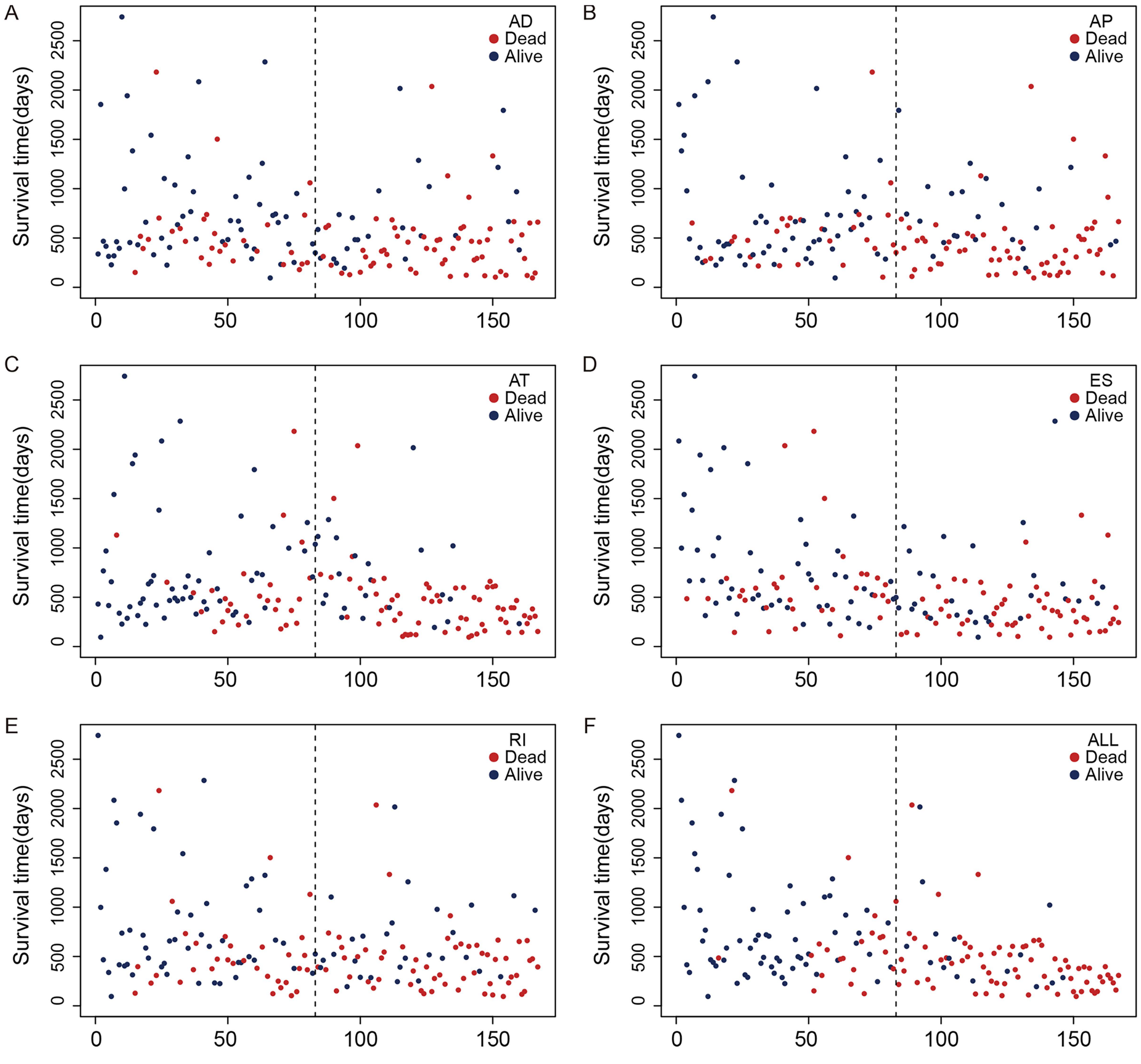
A
B
C
D
E
F
AD
AP
AT
ES
RI
ALL
Dead
Alive
Survival time(days)
0
500
1000
1500
2000
2500
50
100
150

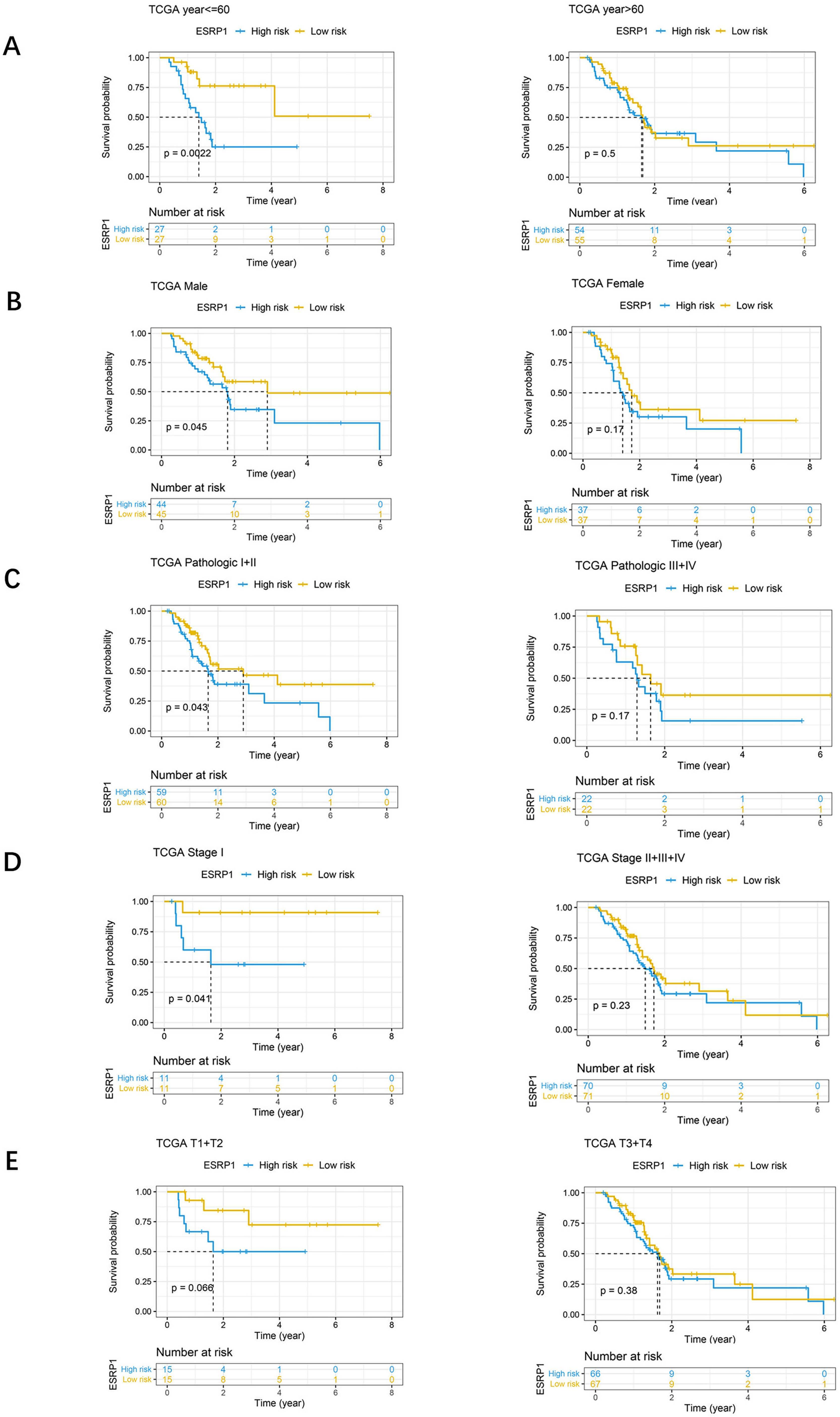
A
TCGA year<=60
ESRP1 High risk Low risk
Survival probability
p = 0.0022
Time (year)
Number at risk
High risk 27 2 1 0 0
Low risk 27 9 3 1 0
TCGA year>60
p = 0.5
High risk 54 11 3 0
Low risk 55 8 4 1
B
TCGA Male
p = 0.045
High risk 44 7 2 0
Low risk 45 10 3 1
TCGA Female
p = 0.17
High risk 37 6 2 0 0
Low risk 37 7 4 1 0
C
TCGA Pathologic I+II
p = 0.043
High risk 59 11 3 0 0
Low risk 60 14 6 1 0
TCGA Pathologic III+IV
p = 0.17
High risk 22 2 1 0
Low risk 22 3 1 1
D
TCGA Stage I
p = 0.041
High risk 11 4 1 0 0
Low risk 11 7 5 1 0
TCGA Stage II+III+IV
p = 0.23
High risk 70 9 3 0
Low risk 71 10 2 1
E
TCGA T1+T2
p = 0.066
High risk 15 4 1 0 0
Low risk 15 8 5 1 0
TCGA T3+T4
p = 0.38
High risk 66 9 3 0
Low risk 67 9 2 1

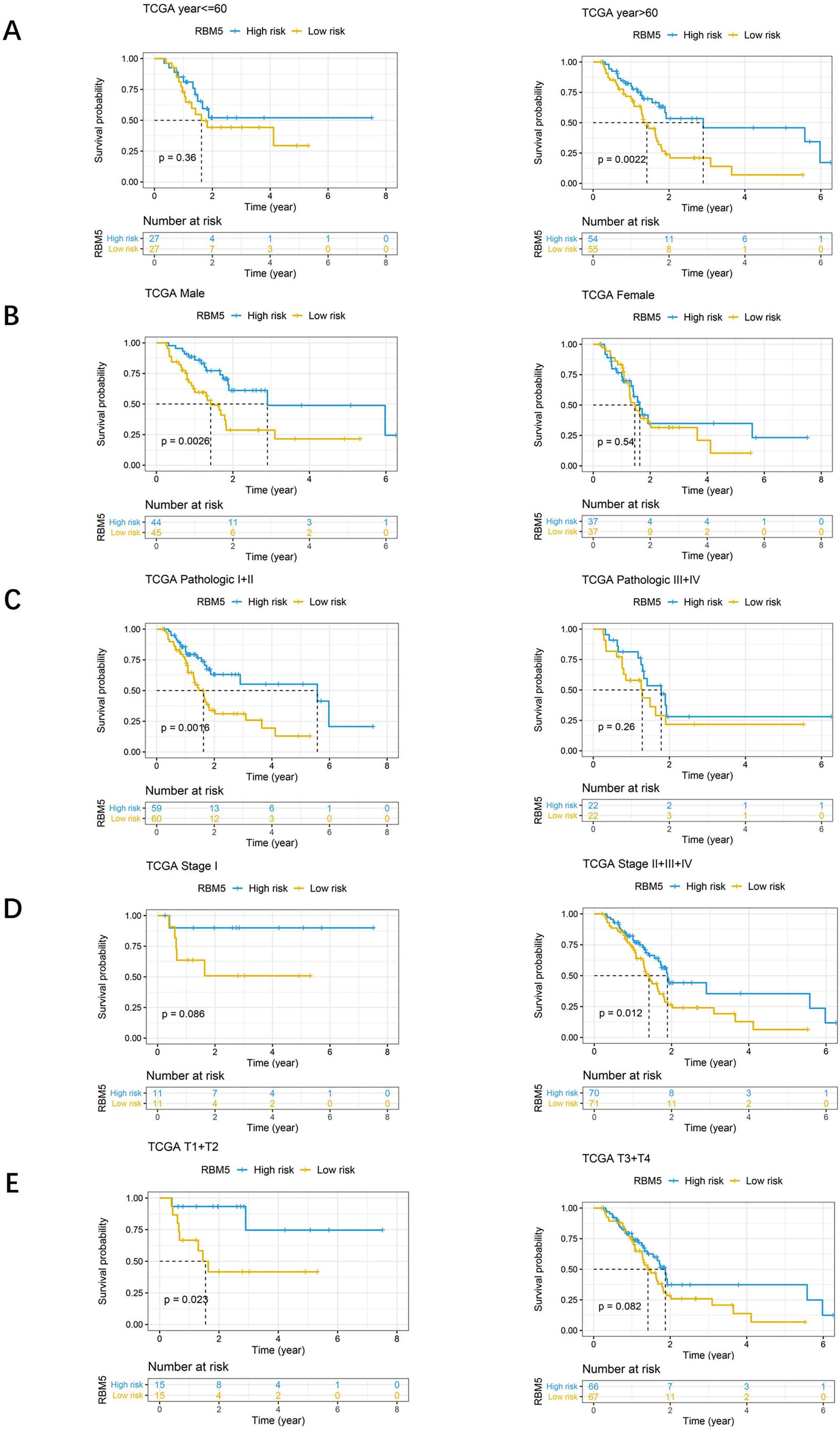
A
TCGA year<=60
RBM5 High risk Low risk
Survival probability
Time (year)
p = 0.36
Number at risk
High risk 27 4 1 1 0
Low risk 27 7 3 0 0
TCGA year>60
p = 0.0022
High risk 54 11 6 1
Low risk 55 8 1 0
B
TCGA Male
p = 0.0026
High risk 44 11 3 1
Low risk 45 6 2 0
TCGA Female
p = 0.54
High risk 37 4 4 1 0
Low risk 37 9 2 0 0
C
TCGA Pathologic I+II
p = 0.0016
High risk 59 13 6 1 0
Low risk 60 12 3 0 0
TCGA Pathologic III+IV
p = 0.26
High risk 22 2 1 1
Low risk 22 3 1 0
D
TCGA Stage I
p = 0.086
High risk 11 7 4 1 0
Low risk 11 4 2 0 0
TCGA Stage II+III+IV
p = 0.012
High risk 70 8 3 1
Low risk 71 11 2 0
E
TCGA T1+T2
p = 0.023
High risk 15 8 4 1 0
Low risk 15 4 2 0 0
TCGA T3+T4
p = 0.082
High risk 66 7 3 1
Low risk 67 11 2 0

Clinical/Pathological Features	Case
**Gender**	
male	93
female	78
**Age at diagnostic**	
≥65	93
＜65	78
**Pathology T**	
T1	7
T2	23
T3	137
T4	4
**Pathology N**	
N0	48
N1	236
uncertain	1
**Pathology TNM**	
IA	6
IB	12
IIA	30
IIB	111
III	4
IV	5
uncertain	3
**Tumor grade**	
G1	28
G2	93
G3	45
G4	2
uncertain	3

Supplementary Table S1: Clinical characteristics of the patients with pancreatic cancer.

Gene symbol	SF	P-value
ESRP1	ESRP1	0.001764
RBM5	RBM5	0.003117
TRA2A	HTra2alpha	0.004274
HNRNPL	hnRNP L	0.00451
HNRNPA0	hnRNP A0	0.00521
SF1	SF1	0.005565
HNRNPM	hnRNP M	0.007592
ELAVL2	HuB	0.021128
RBMX	hnRNP G	0.021606
RBM4	RBM4	0.024785
HNRNPU	hnRNP U	0.029977
SFPQ	PSF	0.036101
YBX1	YB-1	0.037203
NOVA1	Nova-1	0.041152
ELAVL4	HuD	0.043368

Supplementary Table S2: 15 splicing factors were significantly associated with overall survival of pancreatic cancer ($p < 0.05$).
